# Bare Eye Detection of Bacterial Enzymes of *Pseudomonas aeruginosa* with Polymer Modified Nanoporous Silicon Rugate Filters

**DOI:** 10.3390/bios12121064

**Published:** 2022-11-22

**Authors:** Qasim Alhusaini, Walter Sebastian Scheld, Zhiyuan Jia, Dipankar Das, Faria Afzal, Mareike Müller, Holger Schönherr

**Affiliations:** 1 Physical Chemistry I and Research Center of Micro and Nanochemistry and (Bio)Technology (Cμ), Department of Chemistry and Biology, School of Science and Technology, University of Siegen, Adolf-Reichwein-Str. 2, 57076 Siegen, Germany; 2 Department of Chemistry, SRM Institute of Science and Technology, SRM Nagar, Potheri, Kattankulathur 603203, India

**Keywords:** biodegradable polymer, bacterial enzyme, nanoporous silicon rugate filter, poly(lactic acid), *Pseudomonas aeruginosa*, bacteria detection

## Abstract

The fabrication, characterization and application of a nanoporous Silicon Rugate Filter (pSiRF) loaded with an enzymatically degradable polymer is reported as a bare eye detection optical sensor for enzymes of pathogenic bacteria, which is devoid of any dyes. The nanopores of pSiRF were filled with poly(lactic acid) (PLA), which, upon enzymatic degradation, resulted in a change in the effective refractive index of the pSiRF film, leading to a readily discernible color change of the sensor. The shifts in the characteristic fringe patterns before and after the enzymatic reaction were analyzed quantitatively by Reflectometric Interference Spectroscopy (RIfS) to estimate the apparent kinetics and its dependence on enzyme concentration. A clear color change from green to blue was observed by the bare eye after PLA degradation by proteinase K. Moreover, the color change was further confirmed in measurements in bacterial suspensions of the pathogen *Pseudomonas aeruginosa* (PAO1) as well as in situ in the corresponding bacterial supernatants. This study highlights the potential of the approach in point of care bacteria detection.

## 1. Introduction

With increasing occurrences of multidrug-resistant (MDR) bacteria [1,2], new methods of targeted diagnostics and therapy are the focus of research activities worldwide. Despite the development of ultrasensitive detection and identification techniques, such as mass spectrometry-based approaches [3], the vast plethora of challenges cannot be satisfactorily addressed. This is in part due to the wide range of scenarios in which bacteria and bacterial infections should be ideally detected and analyzed. These scenarios differ considerably depending on the area of application, e.g., screening of food, medical products, a patient before entering medical facilities or infected patients, and on the local setting, which may allow access to adequately equipped laboratories or may be, on the contrary, devoid of electricity. Independent of the focus and local challenges, the development of novel cheap, rapid, easy-to-use point of care (PoC) bacteria detection methods may contribute to overcoming the limitations of conventional methods, especially regarding the specific needs for certain application areas and settings.

Specifically, wound infections caused by bacteria are considered to constitute a major healthcare issue [4]. Therefore, the simple detection of pathogenic bacteria that are relevant in this context is highly desirable. *Pseudomonas aeruginosa (P. aeruginosa)*, as a typical Gram-negative pathogenic bacterium, is the cause of many infectious diseases, such as pneumonia, urinary tract infections (UTIs), and bacteraemia [5,6]. The early detection of *P. aeruginosa*, e.g., in wound fluid is critical for determining a suitable treatment before disease progression. Other relevant areas refer to *P. aeruginosa* biofilms in patients that suffer from cystic fibrosis [7].

In general, a multitude of techniques has been used for bacteria detection in a laboratory setting, such as approaches based on polymerase chain reaction (PCR) [8,9], conventional bacterial culture [10], or enzyme-linked immunosorbent assays (ELISAs) [11] and mass spectrometry [3]. However, these techniques cannot be used to detect bacteria outside the laboratory environment, since they require, in addition to those sophisticated facilities, laborious protocols, trained personnel or specialized equipment. To address this need, it is desirable to investigate and develop simple label-free biosensors for the rapid detection of bacteria with high sensitivity.

In earlier work, the detection of *P. aeruginosa* has been demonstrated, e.g., with sensing materials, such as bacteria-secreted virulence factors that open reporter liposomes [12] and hydrolytic enzymes that attack polymeric nanocapsules [13]. These autonomously sensing materials, which also include hydrogels [14,15,16] and swab-type tests [17], produce a color change that can be readily detected by the bare eye.

Another strategy relies on nanoporous materials, such as anodic aluminum oxide (AAO) based sensors [18]. The AAO-based sensing of bacterial enzymes was demonstrated in spiked wound fluid; however, the utilized Reflectometric Interference Spectroscopy (RIfS) is not readily adapted to PoC settings devoid of electricity and controlled temperatures.

In general, the high porosity of nanoporous materials makes them prime candidates for sensing with adequately high sensitivity. In this context, porous silicon (pSi) is a particularly promising nanomaterial for sensing because of its biocompatibility [19,20], tunable nanoporous structure [21,22] and optical properties [23,24]. Several reports have been published in which pSi was utilized as a transducer surface to fabricate label-free biosensors [25,26]. For instance, Gooding and co-workers reported the use of pSi as a host material for monitoring the activity of the enzyme subtilisin with a limit of detection (LOD) of 0.37 pM [27]. Initially, gelatin was immobilized in pSi by a multistep covalent coupling protocol. In addition, the secretion of matrix metalloprotease 9 from stimulated human macrophages was detected. Segal and co-workers have demonstrated the possibility of using pSi sensor for the detection of captured bacterial cells [28]. They fabricated a pSi biosensor to capture *Escherichia coli* cells on the pSi top surface via antibody–antigen interactions. These examples have in common that a complicated multistep functionalization protocol has to be applied, and the detection works via RIfS. The principle of RIfS sensing with porous silicon is the change in the effective refractive index triggered by a change in the molecular species in the pSi matrix [29,30].

Other recent work addressed the sensing of lactoglobulin [31] and DNA with pSi-based photonic materials [32]. Further, Sailor and co-workers developed a biosensor where enzyme-degradable proteins (Zein) were spin-coated onto the pSi surface [33]. A red shift in the reflectivity spectrum was observed during the degradation of the Zein layer by pepsin, attributed to the infiltration of digested products into the pores. Alternatively, electrochemical detection protocols may be applied with adequately modified pSi [34].

In the wider context, however, photonic materials are highly attractive due to the absence of any chromophore for obtaining a strong color response; hence, no bleaching occurs if the sensors are irradiated (which results in vastly increased shelf-life), and no irritating and potentially toxic dyes are being released. PSi photonic structures, which are fabricated electrochemically to produce periodic nanostructures with alternating high and low porosities [35], possess a so-called “stop bandgap” that limits reflected light to specific wavelengths [36]. The resultant peak is very sensitive to the changes in the refractive index of the photonic structure, i.e., the material inside the nanopores. Porous Silicon Rugate Filters (pSiRF) display a sharp and narrow peak in the reflectivity spectrum, which improves the sensitivity of the pSi biosensor. For example, Voelcker and coworkers used pSiRF as a platform for sensing insulin using surface-attached aptamer peptides [37]. The sensing approach was based on the change in the effective refractive index in the pores due to the binding of insulin to the aptamer. Gupta et al. followed the degradation of gelatin for the detection of the enzyme subtilisin [38].

**Scheme 1:**
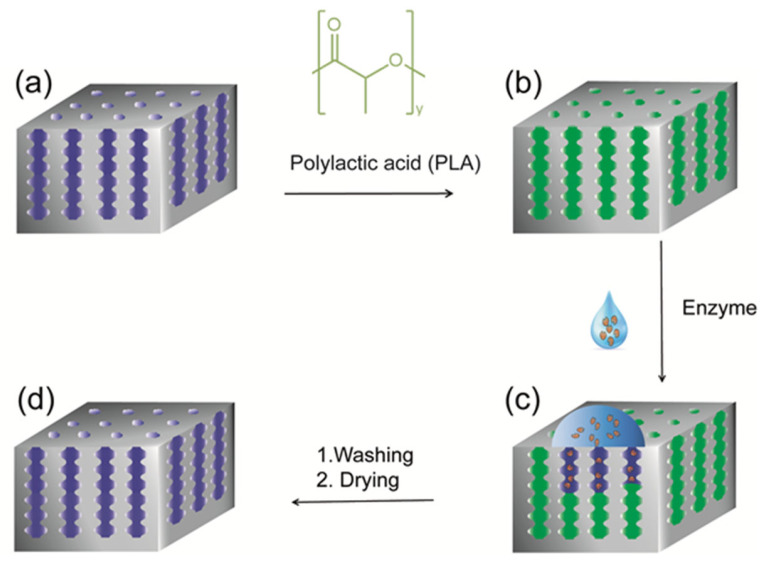
Schematic of the pSiRF-based sensor for bacterial enzymes: (**a**) neat nanoporous pSiRF; (**b**) pSiRF after deposition of PLA inside the pores; (**c**) enzymatic degradation of PLA inside the pores; (**d**) pSiRF after the reaction. The color change of the pSiRF from blue (bare) to green (pores modified with PLA) is due to the increase in the effective refractive index of the sensor caused by the compositional change inside the pores. The original color is recovered after enzymatic degradation of PLA.

Here we report on the investigation and development of a photonic material-based sensor for the bare-eye detection of bacterial enzymes of *P. aeruginosa* (Scheme 1). Exploiting pSiRF as an optical transducer and the enzyme labile polymer poly(lactic acid) (PLA) [39,40,41], the enzyme proteinase K and enzymes secreted by *P. aeruginosa* were detected, providing a visual color change that is discerned by bare eye detection. This approach may afford a simple and direct method to detect *P. aeruginosa* through the enzymatic activity of bacterial enzymes secreted by *P. aeruginosa*, in the absence of other PLA-degrading enzymes.

## 2. Materials and Methods

p-type silicon wafers with an <100> orientation (resistivity 0.01–0.02 mΩ cm) were purchased from Siegert Wafer, Aachen, Germany. Hydrofluoric acid (48% aqueous) was obtained from VWR Chemicals, Darmstadt, Germany. PLA (*M_w_* = 18,000–24,000 g mol^−1^), proteinase K from tritirachium album (≥30 units mg^−1^), acetone (99.8%), ethyl acetate (99.7%), hexane (99%), isopropanol (99.5%), ethylene glycol (99%) and tris(hydroxymethyl)aminomethane were purchased from Sigma Aldrich, Steinheim, Germany. Sodium hydroxide (98.8%) was purchased from ChemSolute, Renningen, Germany. *P. aeruginosa PAO1* (ATCC 15692, isolated from infected wounds) [42,43]). Luria–Bertani medium (LB broth), LB agar, methanol (99.9%), chloroform (99%) and ethanol (96%) were purchased from Carl Roth, Karlsruhe, Germany. Milli-Q water was obtained from Millipore Direct Q 8 system (MILLIPORE, Schwalbach, Germany) with a resistivity of 18 MΩ cm.

### 2.1. Fabrication of pSiRF

The pSiRF structures were etched into highly-doped Si wafers by an anodic electrochemical method [44]. A controllable current source (digital source meter model 2400, Keithley, Cleveland, OH, USA) was used to supply the desired current. The Si wafer was first washed with ethanol and Milli-Q water and then dried in a stream of nitrogen. Afterwards, the pre-cleaned Si wafers were oxidized using a UV/ozone cleaner (Procleaner^TM^, Bioforce Nanosciences, Utah, USA). The electrochemical etching was performed in a homemade Teflon cell with a 2 × 2 cm^2^ piece of Si used as an anode, and a circular Pt wire immersed in the HF solution (see below) acted as a cathode. *(Caution: HF is highly toxic and contact with the skin must be prevented. Adequate safety measures must be taken.)* [35]. To remove the surface contaminations, an initial sacrificial etch was performed at constant current density (55 mA cm^−2^) for 30 s in HF/EtOH 1:2 (*v*/*v*) [29]. Afterwards, the sacrificial layer was dissolved by dipping the specimens into 0.5 M aqueous sodium hydroxide (NaOH) solution, followed by rinsing with Milli-Q water and ethanol. The pSiRF sensors were prepared by application of a sinusoidal current density–time profile (minimum current density: 15 mA cm^−2^ and maximum current density: 50 mA cm^−2^, a period of 7 s was repeated 100 times). Afterwards, the sensors were thoroughly rinsed with ethanol and dried in a stream of nitrogen. Freshly etched sensors were stored under a vacuum (≤10 mbar, 4 h). Then, the sensors were thermally oxidized at 600 °C for 1 h in the air in a muffle furnace (Thermo Fisher, Waltham, MA, USA) with a heating ramp of 10 °C min^−1^ from/to ambient for both the heating and cooling phases [45].

### 2.2. Modification of pSiRF with PLA

The pre-oxidized pSiRF was modified with PLA by a droplet evaporation method. At first, 150 µL of PLA solution (4 wt% in chloroform) was dropped from a pipette onto a pSiRF followed by drying at ambient temperature (≈23 °C) for 4 h. The PLA layer, which remained on the top of the pSiRF sensor, was removed by carefully wiping the sensor surface three times with a paper tissue (Kimberly-Clark) soaked with chloroform under a fume hood. Afterwards, the PLA-modified pSiRF was dried in a stream of nitrogen and was kept in a desiccator at 5 mbar for 2 h. Finally, it was heated on a hot plate at 70 °C for 1 min and allowed to cool to ambient temperature.

### 2.3. Enzymatic Degradation of PLA with Proteinase K

The PLA-modified pSiRF sensors were placed in a 6-well plate containing 300 µL of Tris-HCl buffered proteinase K solution (pH 8.5) with varying concentrations (0.10, 0.25, 0.50 and 1.00 mg mL^−1^). After 0, 30, 60, 120 and 240 min, the reacted sensor was washed with Milli-Q water and dried in a stream of nitrogen and kept in the desiccator at 5 mbar for 2 h, followed by RIfS analysis. For in situ RIfS measurements, the PLA-modified pSiRF was inserted in a homemade flow cell (volume 0.3 mL), and the proteinase K or Tris-HCl buffer solutions were injected.

### 2.4. Bacteria Tests

One single colony of *P. aeruginosa* (PAO1) was transferred from an agar plate to a 15 mL reaction tube within 5 mL LB medium and then incubated under 200 rpm for 24 h at 37 °C. The absorbance (Abs) of the resulting bacterial suspension was measured by UV−Vis spectroscopy (see below) at λ = 600 nm in a well of the transparent 96-well plates containing 100 µL of the bacterial suspension. Afterwards, the bacterial suspension was diluted to Abs_600_ = 0.8 with fresh LB medium. Next, 200 µL of the diluted bacterial suspension with Abs_600_ = 0.8 was transferred to 50 mL reaction tubes already containing 20 mL of LB medium and further incubated under 200 rpm for another 24 h at 37 °C for the growth of *P. aeruginosa*. A 0.2 μm syringe filter (CME) was used to remove all the bacteria, and the resultant sterile supernatant was used for further analysis. In the end, 3 mL of resulting bacterial suspension after 24 h incubation was added into the well of the transparent 6-well plates containing a PLA film-coated pSiRF, followed by further incubation for 24 h at 37 °C. A part of the suspension was diluted and spread out onto an LB agar plate and incubated at 37 °C overnight to determine the bacterial colony forming units mL^−1^ (CFU mL^−1^) of the inoculums. The PLA-modified pSiRF was removed from the bacterial suspension or LB medium and washed gently with LB medium and Milli-Q water. Finally, the pSiRF was dried in a laminar flow cabinet for 1 h. For in situ analysis, PLA-coated pSiRF sensors were inserted in a homemade flow cell (volume 0.3 mL) and incubated in LB medium or supernatant solution.

### 2.5. UV−Visible Spectroscopy

UV−Vis spectroscopy measurements were carried out on a microplate reader (Tecan SAFIRE, Tecan, Switzerland) at 25 °C. The absorbance of the cultured bacterial suspension at 600 nm was recorded using a 96-well plate (transparent, polystyrene, flat bottom, Sarstedt, Germany) as a sample holder with a clear viewseal sealer (Greiner Bio-One, Kremsmünster, Austria).

### 2.6. Reflectometric Interferometry Spectroscopy (RIfS)

RIfS spectra were measured on pSiRF sensors before and after PLA modification at ambient temperature [18,46,47]. The pSiRF sensor was fixed into a homemade flow cell and shined with white light at a normal incident angle using a tungsten halogen (LS-1) through one arm a bifurcated optic fiber. The reflected light was collected through the other arm of a bifurcated fiber using a charge-coupled device (CCD) detector (USB-2000+VIS-NIR-ES). For online measurements, the flow cell was connected to a peristaltic pump (Ismatec, Germany), and the Tris-HCl buffer or proteinase K solutions were injected at a flow rate of 0.3 mL min^−1^; after 4 min the pump was stopped but the measurement was continued to the desired time. The measurements were recorded by the software SpectraSuite (Ocean Optics). Ten scans were averaged for each measured data point. The data were analyzed using the Igor program (Wavemetrics Inc., Oregon, USA).

### 2.7. Field Emission Scanning Electron Microscopy (FESEM)

For the characterization of the morphology of the pSiRF sensors, FESEM micrographs were acquired on a Zeiss Ultra 55cv Field Emission Scanning Electron Microscopy (FESEM) (Zeiss, Oberkochen, Germany). The operation voltage for all the measurements was 10 kV, and the detector was the Inlens secondary electron. The FESEM micrographs were analyzed using ImageJ software (v1.50d). Elemental analysis via energy-dispersive X-ray spectroscopy (EDX) was also performed, and the data were analyzed using the software NSS.

## 3. Results

### 3.1. Fabrication of pSiRF Sensors

**Figure 1:**
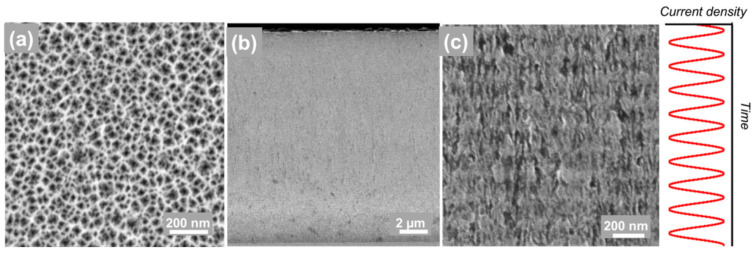
FESEM images of pSiRF sensor: (**a**) Top view; (**b**,**c**) cross-sectional views with low and high magnification, respectively, showing the modulated pore diameters. The schematic temporal current density profile corresponds to the data in panel (**c**).

pSiRF sensors were prepared via electrochemical etching of p-type Si (100) wafers in ethanolic hydrofluoric acid by modulating the current density in a sinusoidal manner. The morphology of a typical pSiRF sensor after the subsequently applied thermal oxidation process [48] is shown in the top view FESEM micrograph in Figure 1a. This image shows nanopores that are randomly distributed across the surface. The mean pore diameter was 12 ± 5 nm, determined from the top view image using ImageJ software (Figure S1, Supporting Information). The average porosity was obtained by analyzing the FESEM images and by the Spectroscopic Liquid Infiltration Method (SLIM) [46]. In SLIM, the effective optical thickness is measured by RIfS before and after ethanol has infiltrated the pores and displaced the air inside. The values of the porosity thus determined were 45 ± 5% and 44 ± 4% by FESEM and SLIM, respectively. The thickness of the sensor was 14.6 ± 0.5 μm, which was also determined from the analysis of cross-sectional FESEM images (Figure 1b). The magnified image shown in Figure 1c reveals a periodic nanostructure with a spacing of 148 ± 3 nm, which is oriented perpendicular to the surface of the pSiRF. The total thickness of the pSiRF structure is consistent with the thickness of one layer (148 ± 3 nm) and the number of periodic repeats (100 times).

### 3.2. Stabilization of pSiRF Sensors

**Figure 2:**
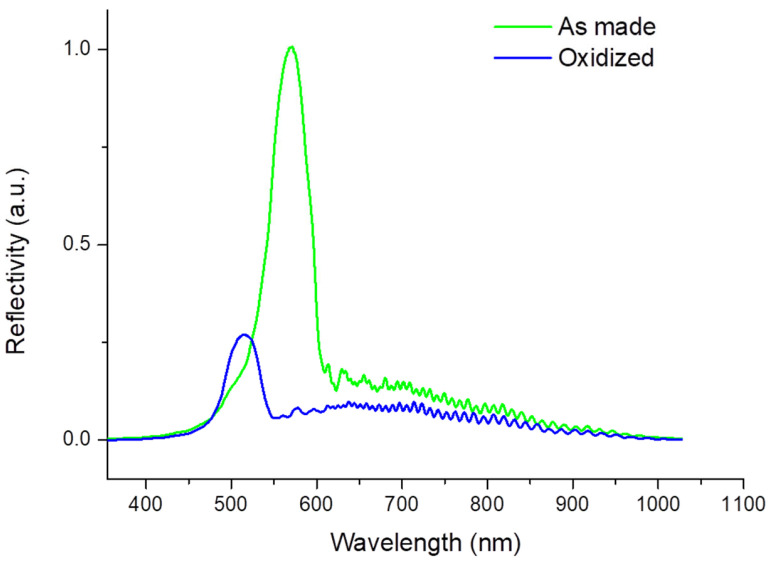
RIfS spectra of pSiRF in air at 23 °C before (green) and after (blue) thermal oxidation in air at 600 °C for 1 h.

In the current study, all pSiRF sensors were thermally oxidized at 600 °C for 1 h in air, as it was shown to be a suitable protocol to stabilize pSi for biological applications [25,47]. The RIfS spectra (Figure 2) showed an obvious blue shift in the peak position from 570 nm to 513 nm as a result of the thermal treatment on the optical properties of the freshly prepared pSiRF. This blue shift of 57 nm is due to the transformation of Si to SiO_2_, which results in a reduction in the refractive index from 3.5 for Si to 1.4 for amorphous SiO_2_ [47]. Therefore, the shift in the reflectance spectrum upon thermal treatment is consistent with the formation of SiO_2_, in full agreement with previous studies [48,49].

### 3.3. Modification of pSiRF with PLA

PLA was loaded into pSiRF pores by placing a droplet of PLA solution onto the surface of the sensors, which is allowed to evaporate. As shown in Figure S2, the PLA film covering the pores was successfully removed by cleaning the top surface with chloroform-soaked tissue. Subsequently, most of the pSiRF pores remained open after coating with PLA.

**Figure 3:**
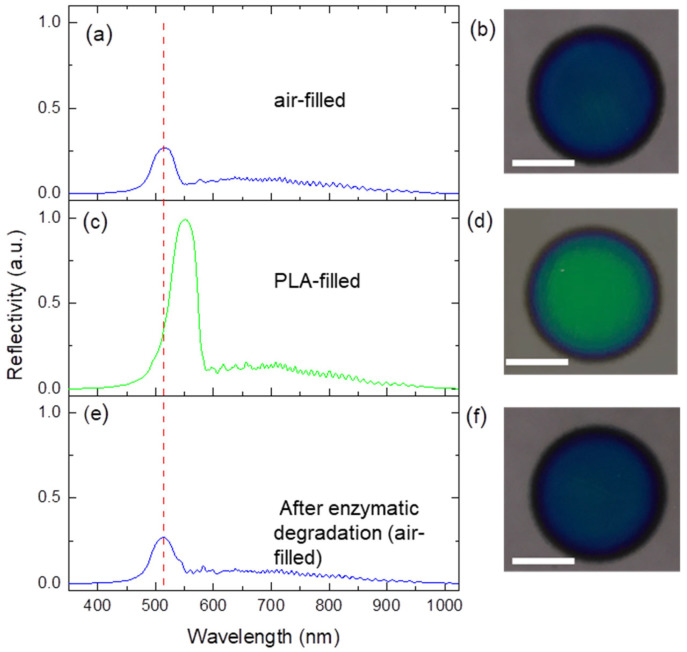
RIfS spectra and photographs of dried pSiRF: (**a**,**b**) air-filled pSiRF, (**c**,**d**) PLA-filled pSiRF and (**e**,**f**) pSiRF after enzymatic degradation of PLA with proteinase K (scale bars = 0.5 cm).

Similarly, the PLA-filled pSiRF nanopores exhibited a shift in the RIfS spectra compared to the neat air-filled pSiRF sensors. The position of the peak in the reflectance spectrum of the empty (air-filled, before PLA deposition) pSiRF was centered at 513 nm (Figure 3a). The fringes in the reflectivity spectrum are caused by the interference of reflected light at the Si–SiO_2_ (bottom side) and air–SiO_2_ (top side) interfaces of the pSiRF. Figure 3b shows a photograph of the empty pSiRF, which shows blue color in reflection. After PLA deposition, the peak (Figure 3c) was red-shifted by 37 ± 2 nm due to an increase in the effective refractive index (*n_eff_*) of the pSiRF. Additionally, the intensity of the peak increased after PLA modification, which is due to the refractive index contrast at the interfaces (e.g., from air to polymer). This was also observed in the literature [50]. The color changed to green (Figure 3d). After enzymatic degradation, the original spectra and color were regained (Figure 3e,f).

The *n_eff_* values of the empty and PLA-modified pSiRF can be determined using the Bragg–Snell law (Equation (1)) [51,52].

(1) $$m\lambda =2Ln_{\mathrm{eff}}-\cos\theta \;$$

where *m* is the interference order, λ is the wavelength, *L* is the periodic thickness obtained from the FESEM images (148 nm) and *θ* is the incidence angle of light (90°).

Knowing the position of the peak before and after PLA deposition in SiRF (Figure 3a vs. Figure 3c), the values of *n_eff_* were determined to be 1.73 and 1.96 for empty pSiRF (air-filled) and PLA-filled pSiRF, respectively. The increase in *n_eff_* after PLA infiltration in pSiRF is due to the replacement of air, which has a small refractive index (1.000) [53], and with the polymer PLA, which possesses a high refractive index (1.459) [54]. The Bruggeman effective-medium approximation was used to predict how much the spectral position of the stop-band peak would shift if the polymer infiltrates the pores of pSiRF (Equation (2)) [55].

(2) $$P\;\frac{n^{2}{}_{\mathrm{void}}-n^{2}{}_{eff}}{n^{2}{}_{\mathrm{void}}+2n^{2}{}_{eff}}+\left(1-P\right)\frac{n^{2}{}_{{\mathrm{SiO}}_{2}}-n^{2}{}_{eff}}{n^{2}{}_{{\mathrm{SiO}}_{2}}+2n^{2}{}_{eff}}=0\;$$

where P is the porosity, $n_{\mathrm{void}}$ is the refractive index of the medium filling the pores and $n_{{\mathrm{SiO}}_{2}}$ is the refractive index of the ${\mathrm{SiO}}_{2}\;$ that makes up the porous structure [35].

If the pores are completely occupied with PLA, the peak of empty pSiRF shifts from 513 nm to 580 nm, according to Equation (2). However, the observed shift of 37 nm in the reflectance peak in the PLA-modified pSiRF is smaller; hence, the PLA did not fill the pores completely: the pore filling was 55%. It is worth mentioning that the reflectance spectrum of both PLA-modified pSiRF and empty (air-filled) pSiRF did not show any sign of additional peaks (stop bands) or superposition of RIfS response. Additional or multiple additional peaks in the reflectance spectrum can appear if a modified pSiRF exists as a biphasic system and if PLA fully occupies a part at the bottom/top of pores and the rest remains air-filled [50]. EDX mapping of the carbon signals showed that PLA was loaded successfully into the pores (Figure S3). The pore filling was also determined by thermogravimetric analysis (TGA). Based on TGA data, the pore filling was 54 ± 14 %, which is comparable to the value determined with RIfS (Figure S4).

### 3.4. Enzymatic Degradation of PLA by Proteinase K

As mentioned, the deposition of PLA in pSiRF not only causes a red shift in the spectral position but also leads to color change from blue to green due to the concomitant increase in *n_eff_* of pSiRF (Figure 3d). The PLA-infiltrated pSiRF can, thus, be used to detect the enzyme proteinase K, which is known to cleave the ester bonds of PLA [18,56,57]. The degradation of PLA by proteinase K in the pores should lead to a change in *n_eff_* of the pSiRF. The reflectance spectrum was, hence, recorded after incubation of PLA-modified pSiRF with proteinase K for 7 h. As can be seen in Figure 3e, the position of the peak is blue-shifted to the original position. This observation indicates that the PLA in pSiRF was indeed degraded by proteinase K, and the resulting degraded fragments were removed in the washing step. Figure 3f displays a photograph of the pSiRF, confirming a color change from green to blue, which was readily discernible by the bare eye after PLA degradation.

**Figure 4:**
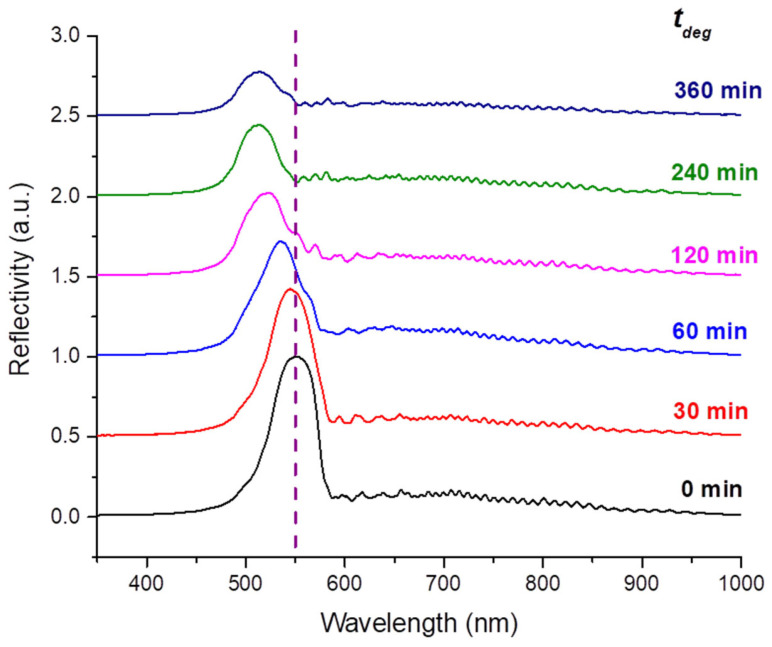
RIfS spectra of PLA coated pSiRF after incubation with 1.00 mg mL^−1^ proteinase K for 0, 30, 60, 120, 240 and 360 min. The RIfS measurement was performed after the washing and drying steps of pSiRF.

This enzymatic reaction was also studied for different degradation times. As shown in Figure 4, the spectrum of the PLA-modified pSiRF shows a peak centered at 550 nm at time zero (*t* = 0). Thirty minutes after starting the enzymatic reaction, the peak was blue-shifted by ~ 6 nm, which is attributed to a decrease in the refractive index after partial removal of PLA from the pores. The peak was blue-shifted by ~37 nm up to 240 min and remained unchanged at longer incubation times, providing 100% degradation of the loaded PLA, and the peak position of unloaded pSiRF was restored. Moreover, the intensity of the peak decreased as the reaction progressed.

Next, the response of the PLA-modified pSiRF sensor was investigated for different enzyme concentrations. As shown in Figure S5, Supporting Information, the shift in the peak positions obtained after applying proteinase K to the PLA-modified pSiRF sensor for a given time exhibited an increase with increasing concentration of the enzyme.

**Figure 5:**
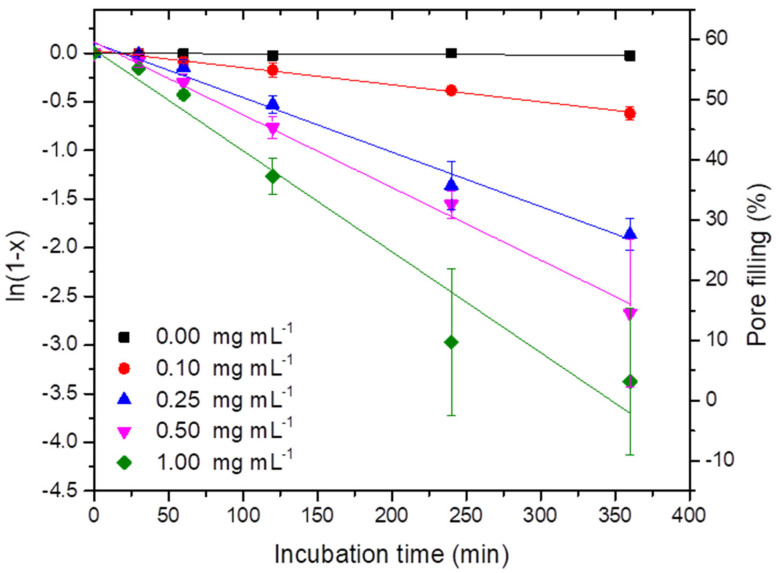
Plot of ln(1 − *x*) (*x*: extent of reaction) and the pore filling as a function of incubation time for the degradation of PLA inside pSiRF in 0.10, 0.25, 0.50 and 1.00 mg mL^−1^ of proteinase K solution at 25 °C for 0, 30, 60, 120, 240 and 360 min. The pore filling was estimated based on Figure S6. The data were presented as mean ± standard deviation. The solid lines correspond to linear least-squares fit of RIfS data. All measurements were conducted in the dried state.

The extent of the reaction *x* can be expressed as a function of incubation time [58].

(3) $$x=\frac{\Delta \lambda_{0}-\Delta \lambda_{t}}{\Delta \lambda_{0}-\Delta \lambda_{\infty }}$$

where Δ*λ*_0_ is the shift in the wavelength shift at time zero, Δλ_t_ at time t and Δλ_∞_ at infinitive time. Figure 5 illustrates the course of the enzymatic degradation ([1 − *x*], with the extent of reaction (*x*) as a function of reaction time, along with the change in the pore filling. The pore filling was estimated from Figure S6 (Supporting Information), as described elsewhere [59,60]. The PLA pore filling was reduced with the incubation time, following the reduction in the refractive index in the pores. Furthermore, Figure 5 shows that the extent of reaction increases substantially with elevated concentration of proteinase K. In contrast, there was no significant response of the sensor in the absence of the proteinase K (Tris-HCl buffer). Hence, since the response is due to the enzymatic reaction and ion addition, we may conclude that the PLA-modified pSiRF is stable in the buffered medium for at least 6 h. The data were least-squares fitted to evaluate the apparent rate constants k.

**Figure 6:**
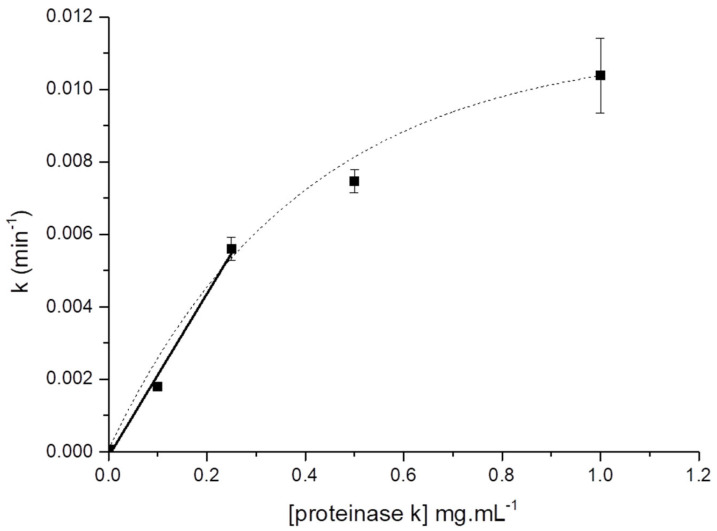
Plot of apparent rate constant (*k*) versus proteinase K concentrations (0.10, 0.25, 0.50 and 1.00 mg mL^−1^). The values for *k* were obtained from linear fits in Figure 5; the error bars denote the standard error from these fits. The solid line corresponds to the linear least-squares fit of the first three data points used to estimate the corresponding slope (0.022 mg mL^−1^ min^−1^) and serves, similar to the dotted line, as a guide to the eyes only.

The *k* values were plotted against the proteinase K concentrations (Figure 6). It was observed that *k* increased linearly for the lower proteinase K concentrations and then levelled off. The later observation is attributed to the limited fraction of exposed PLA inside the nanopores. For higher proteinase K concentration, the amount of PLA became saturated to enzymes for the hydrolysis of ester bonds and, consequently, the rate did not increase further.

Moreover, the kinetics of the enzymatic reaction of PLA deposited on a flat silicon wafer was also studied. The data in Figure S8 (Supporting Information) showed that the thickness of the PLA film decreased linearly with incubation times. It can also be concluded that the degradation rate of PLA on the flat Si wafer was much faster than in pSiRF. The latter can be explained by the availability of a higher amount of PLA in the area of the sensor exposed to the enzyme molecules.

**Figure 7:**
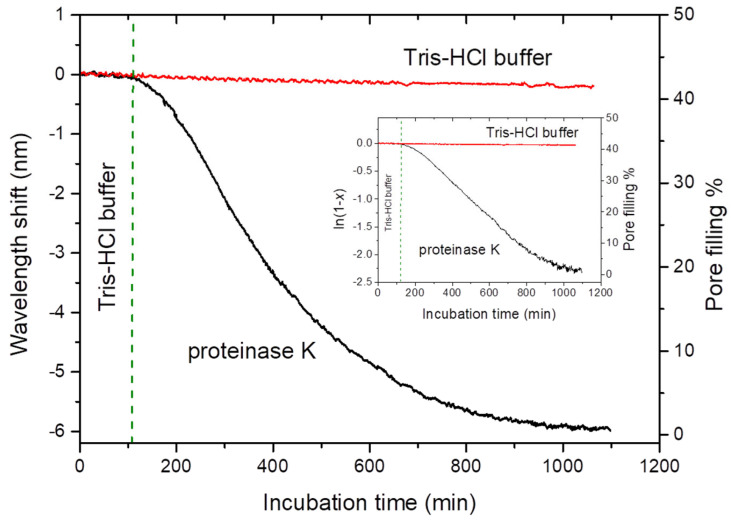
Plot of in situ RIfS measurement of enzymatic reaction in PLA modified pSiRF with proteinase K solution (1.00 mg mL^−1^) and Tris-HCl buffer at 25 °C.

The enzymatic reaction of the PLA-modified pSiRF with proteinase K was also followed under identical conditions as the previous experiment by RIfS. Figure 7 shows two wavelength–incubation time datasets: one is for PLA-modified pSiRF exposed to Tris-HCl buffer (blank) and the other is for PLA-modified pSiRF exposed to proteinase K solution. Before the enzymatic reaction, Tris-HCl buffer was injected over PLA-modified pSiRF for 2 h followed by injecting proteinase K solution.

In contrast to the virtually constant wavelength observed in the blank experiment during 18 h, a blue shift in the wavelength was observed almost instantaneously after injection of the proteinase K. The blue shift in the peak position is due to the reduction in the refractive index of the void in the pores because of the replacement of the PLA by the buffer. A value for *k* of 0.003 min^−1^ was calculated from Figure 7. This value of *k* is three times smaller than that obtained in ex situ measurements (compare Figure 5). This difference points at the importance of sample handling. After each incubation of the sensor in the ex situ experiment, the sensor was taken out of the medium, washed and dried, and then characterized with RIfS. By contrast, no washing or drying steps were applied in the in situ experiment; hence, no convection took place, and degraded PLA may have remained trapped inside the pores.

### 3.5. Enzymatic Degradation of PLA by P. aeruginosa

**Figure 8:**
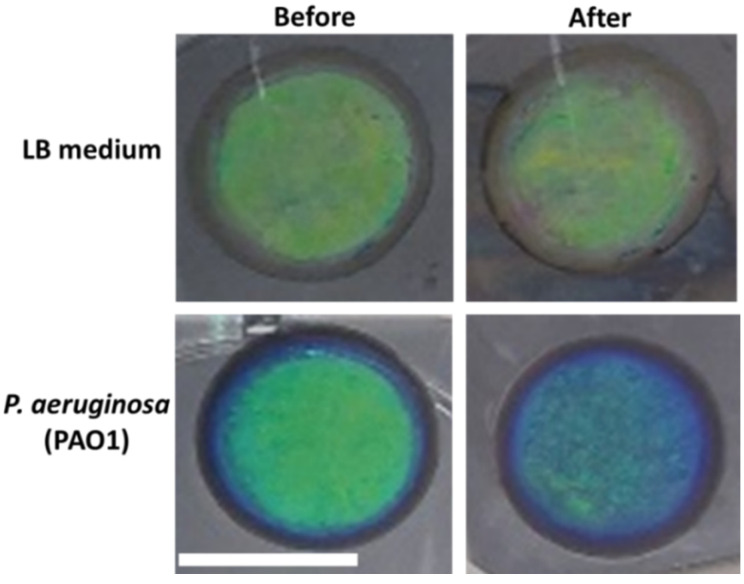
Photographs of the pre-dried PLA-coated pSiRF before and after the immersion in bacteria-free LB medium and in *P. aeruginosa* (PAO1) suspension (bacteria starting concentration: 1.9 × 10^9^ CFU.mL^−1^) in LB medium at 37 °C for 24 h (end concentration: 3.7 × 10^9^ CFU.mL^−1^), scale bar = 1 cm.

The essential step to demonstrate the functionality of bacterial enzyme detection is a test with the living target bacteria. The experiment was performed by immersing the PLA-modified pSiRF sensor directly into a suspension of *P. aeruginosa* (cultured for 24 h) in a six-well plate. The color changes due to the enzymatic reaction were documented with a conventional smartphone camera. As can be seen in Figure 8, a strong color change from green to blue was observed after exposing the PLA-modified pSiRF sensor to the suspension of *P. aeruginosa* in LB medium for 24 h. As a control experiment, a PLA-modified pSiRF sensor was immersed in a bacteria-free LB medium under the same conditions. Here, no significant color change was observed after 24 h. These results indicate that the color change on pSiRF occurs only when the target bacteria are present, which are known to secrete enzymes (proteases) that digest the PLA [61,62].

**Figure 9:**
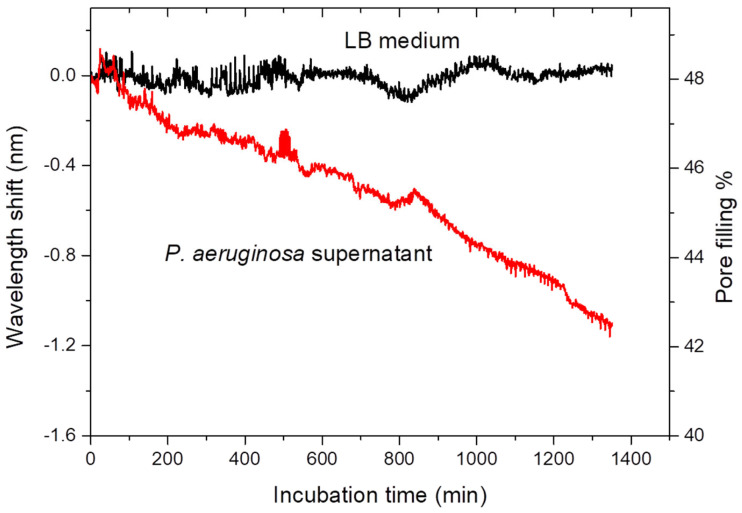
Plot of in situ RIfS measurement during incubation of a PLA-modified pSiRF sensor in sterile *P. aeruginosa* (PAO1) supernatants obtained from a 1.9 × 10^9^ CFU mL^−1^ suspension by filtration (black line) and in pure LB medium (red line). The PLA-modified pSiRF sensor was stable in LB for more than 22 h, whereas incubation in *P. aeruginosa* supernatant caused a quasi-linear blue shift in the wavelength. The spectral positions of the characteristic peaks were corrected by subtracting the wavelength obtained before incubation in the bacterial supernatant or in LB medium.

Finally, the PLA-modified pSiRF sensor was also tested in situ in the corresponding sterile filtered supernatant of *P. aeruginosa* (PAO1). Again, pure LB medium was chosen as a blank. Figure 9 shows the RIfS measurement carried out during the incubation in the sterile bacterial supernatant and in LB medium that have not been in contact with any bacteria, respectively. It was observed that the peak was blue-shifted for the *P. aeruginosa* supernatant, whereas no significant change in the peak position was recorded in LB, even after 22 h of incubation. The results confirm that PLA was stable in the LB medium and degraded in the *P. aeruginosa* (PAO1) supernatant, thus indicating that enzymes secreted by *P. aeruginosa* in this medium can be still detected after removing the bacteria.

Not surprisingly, the results revealed that the reaction was slower compared to the reaction in the bacterial suspension. A possible explanation for these results may be the lower enzyme concentration in the supernatant compared to that in the bacterial suspension due to adsorption to the filters used. Further, it was reported that the filtration process caused an effect on enzyme activity due to the enzyme denaturation and/or the reduction in concentration of enzyme [63,64]. Moreover, the increase in the bacteria concentration (from 1.9 × 10^9^ CFU mL^−1^ to 3.7 × 10^9^ CFU mL^−1^) after 24 h incubation of the PLA-modified pSiRF sensor in bacterial suspension cannot be neglected, which might lead to an enhancement in the concentration of secreted enzymes.

As no additional enzymes were continuously produced in bacterial supernatants like in bacterial suspensions where metabolically active bacteria can still produce fresh enzymes that can be detected in real time, enzyme activity in sterile filtered supernatants can decrease over time due to possible instability of enzymes in LB medium exposed to higher temperatures.

The results underlined the feasibility of this sensing platform to be potentially used as a PoC system for bacterial enzyme detection by the bare eye. The response times and sensitivities are currently inferior compared to other nanocapsule or hydrogel-based approaches [12,13,14,15,16,17], but the sensor design and the materials render this macro(molecule)-nano(porous) platform extremely robust. The color change of the sensor from green to blue may be difficult to discern, depending on the lighting conditions, in a real application, but the colors can be adapted by changing the Rugate structure. Additionally, objective detection utilizing smartphone-based detection is an already demonstrated option [65]. The response time depends on the enzyme concentration, hence, LOD values (not determined here) are time dependent, as discussed before [16]. Moreover, this approach could be employed for real-time detection of secreted enzymes from a wider range of bacteria if PLA is replaced by, e.g., hyaluronan or poly(ε-caprolactone) or other labile polymers specifically prone to degradation by a bacterial enzyme selective for other clinically labile bacteria, such as hyaluronidase or lipases from *Staphylococcus aureus* [34,66,67].

## 4. Conclusions

A PLA-modified pSiRF nanoporous sensor for the bare-eye detection of bacterial enzymes of *P. aeruginosa* is demonstrated for the first time. The macro(molecule)-nano(porous) platform (PLA-modified pSiRF) was successfully fabricated by functionalization of pSiRF with a biodegradable polymer (PLA) through a droplet evaporation method. The role of pSiRF as an optical transducer was ascertained from the significant changes in wavelength in the RIfS spectra and from the prominent changes in the color of the sensor (blue to green) in pure proteinase K solution, *P. aeruginosa* (PAO1) cultures as well as the corresponding sterile culture supernatant. Specifically, a redshift of the wavelength (513 nm to 550 nm) and green color were noticed in the PLA-modified pSiRF, which is due to an increase in the effective refractive index after PLA infiltration into the nanopores of pSiRF. The wavelength can be reverted (from 550 nm to 513 nm) upon enzymatic degradation of PLA, and the sensor became blue. The color change from green to blue could be detected by the bare eye after immersion and drying of the sensor and after incubation with a suspension of viable *P. aeruginosa*. The combined results of ex situ and in situ RIfS measurements, on the one hand, and the visual inspection of the photonic sensors, on the other hand, indicate that this principle may be utilized for sensing bacterial enzymes of clinically relevant bacterial pathogens.

## Data Availability

The data presented in this study are available on request from the corresponding author. The data are not publicly available as they are part of an on-going study.

## Supplementary Materials

The following supporting information can be downloaded at: https://www.mdpi.com/article/10.3390/bios12121064/s1, Supplementary Information: Histogram of the pore diameter distribution; top view of FESEM images of pSiRF before and after PLA deposition; EDX carbon mapping and TGA data of PLA modified pSiRF; RIfS measurements for organic solvents and calculated pore filling data; ellipsometric thickness of PLA films after enzyme treatment on a flat silicon surface; bacterial growth curve based on OD_600nm_ measurement.
