# Supplementary material for: Bare Eye Detection of Bacterial Enzymes of Pseudomonas aeruginosa with Polymer Modified Nanoporous Silicon Rugate Filters
Source: Biosensors (Basel). 2022 Nov 22;12(12):1064. doi: 10.3390/bios12121064 (PMC9776340; doi:10.3390/bios12121064)
Supplement: Supplementary file 1 [file biosensors-12-01064-s001.zip › biosensors-2032678-supplementary.pdf]

Supporting Information

Bare Eye Detection of Bacterial Enzymes of *Pseudomonas aeruginosa* with Polymer Modified Nanoporous Silicon Rugate Filters

Qasim Alhusaini [1], Walter Sebastian Scheld [1], Zhiyuan Jia [1], Dipankar Das [1,2], Faria Afzal [1], Mareike Müller [1] and Holger Schönherr [1,*]

[1] Physical Chemistry I & Research Center of Micro and Nanochemistry and (Bio)Technology (**Cμ**), Department of Chemistry and Biology, School of Science and Technology, University of Siegen, Adolf-Reichwein-Str. 2, 57076 Siegen, Germany

[2] Department of Chemistry, SRM Institute of Science and Technology, SRM Nagar, Potheri, Kattankulathur 603203, India

* Correspondence: schoenherr@chemie.uni-siegen.de, Tel.: (+49)-271-740 2806

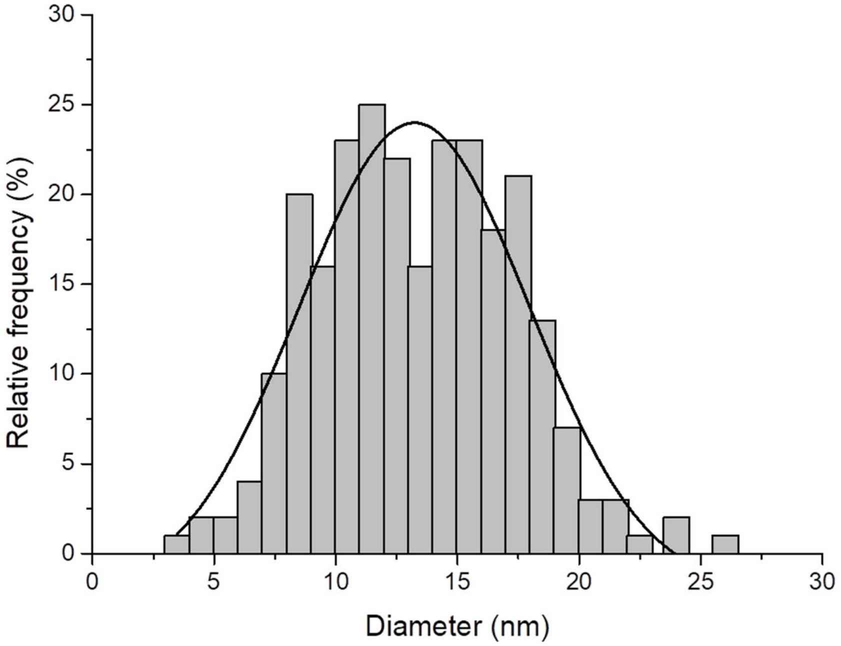

Figure S1. Histogram of pore sizes obtained by analyzing top view FE-SEM images of oxidized pSiRF with ImageJ software.

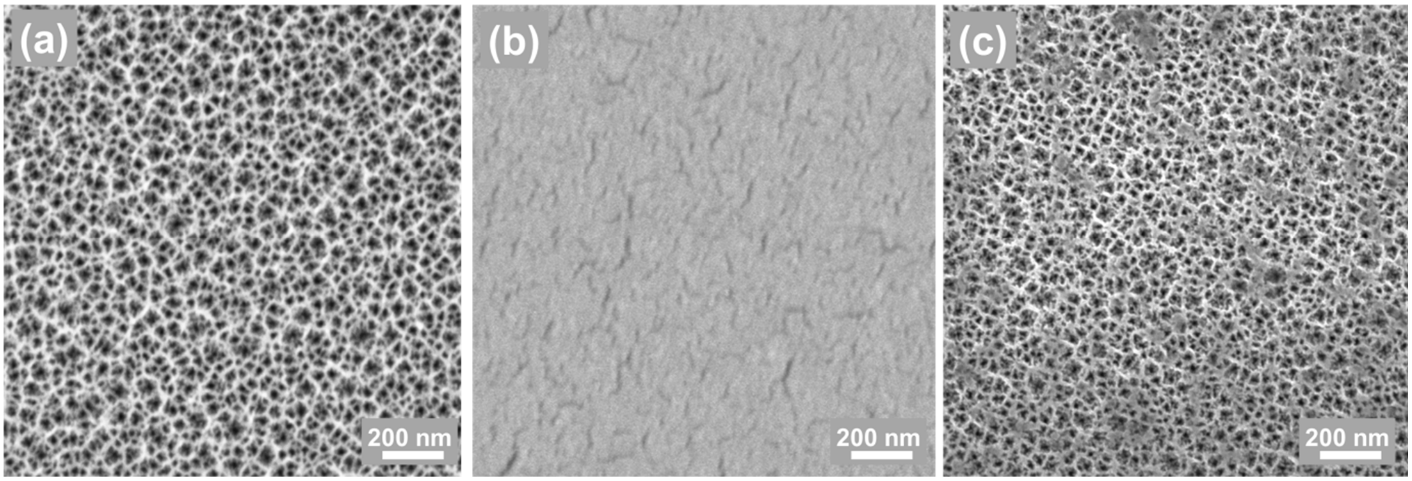

Figure S2. FESEM images of: (a) neat pSiRF, (b) pSiRF after PLA deposition, (c) PLA-coated pSiRF after cleaning of the top surface with a chloroform-soaked tissue.

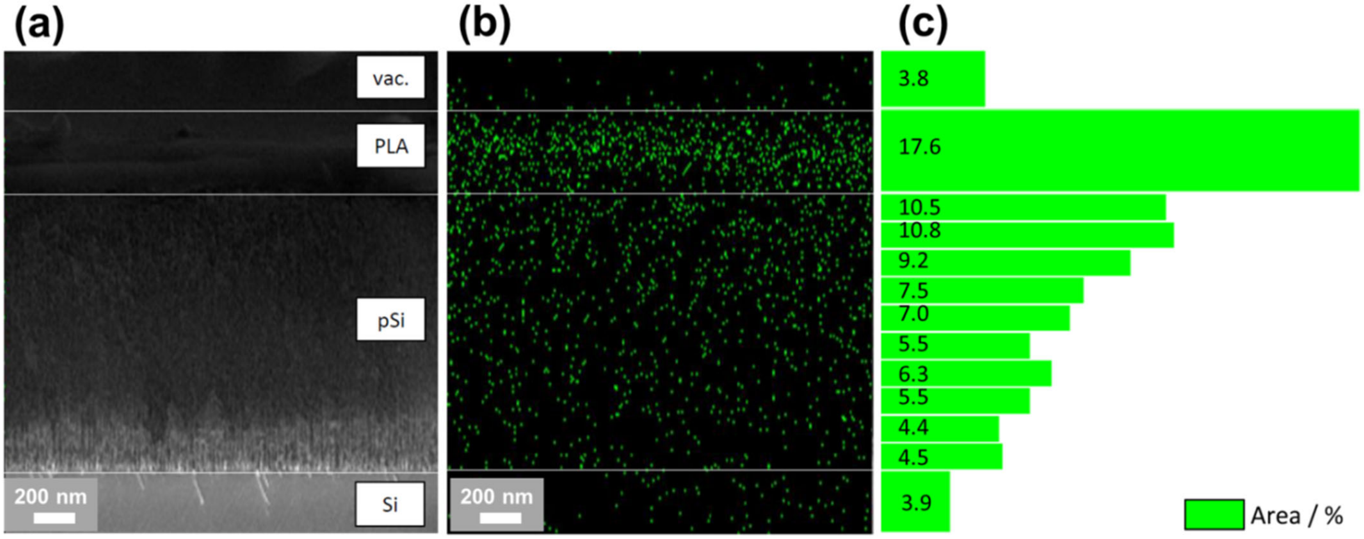

Figure S3. (a) Cross-sectional FESEM image, (b) EDX carbon mapping of PLA-modified pSiRF, (c) measured area (percentage of carbon signal) from graph (b). Different areas were divided with the white bars starting from the top: Vacuum, PLA layer on top of pSi, pSi pores and bulk Si.

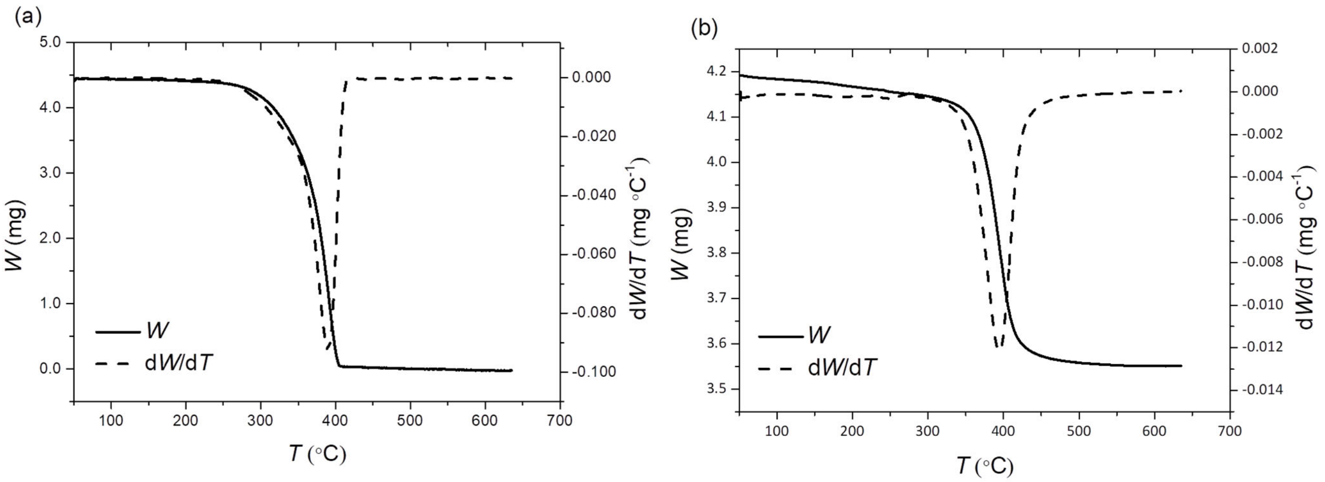

Figure S4. TGA thermograms of PLA (a) in the bulk, (b) inside the pores of pSiRF with an average pore diameter 12 ± 5 and pore length 14.6 ± 0.5 µm. The heating ramp was conducted from 50 to 880 °C with a heating rate of 10 °C per minute in a 15 mL/min Nitrogen gas flow. The terms *W* and d*W*/d*T* are the mass and the mass derivative, respectively.

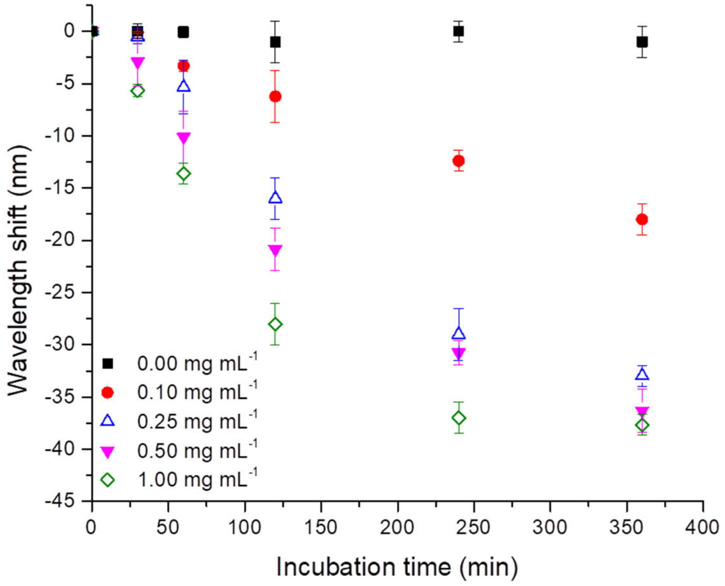

Figure S5. Plot of wavelength shift of four sets of PLA modified pSiRF sensors in the dry state, after incubation in 0.10, 0.25, 0.50, and 1.00 mg mL^{-1} of proteinase K solution for 0, 30, 60, 120, 240 and 360 min. The error bars are the standard deviation (n=3).

The refractive index of the void in the pores and the spectral peak position was studied using a series of organic solvents. These organic solvents were used in previous studies to ascertain how pSi sensors respond to changes in their porous medium [1,2] In our work, methanol, ethanol, ethyl acetate, hexane, isopropanol, and ethylene glycol were used to infiltrate the pores of bare pSiRF and the changes were monitored by RIfS (Figure S6). The refractive indices of the solvents outside the pSiRF pores were measured with an Abbe refractometer. The positions of the peaks observed in RIfS were plotted against the refractive indices of the solvents. Based on a fit of these data, the refractive index of the dried PLA modified pSiRF was calculated (Table S1).

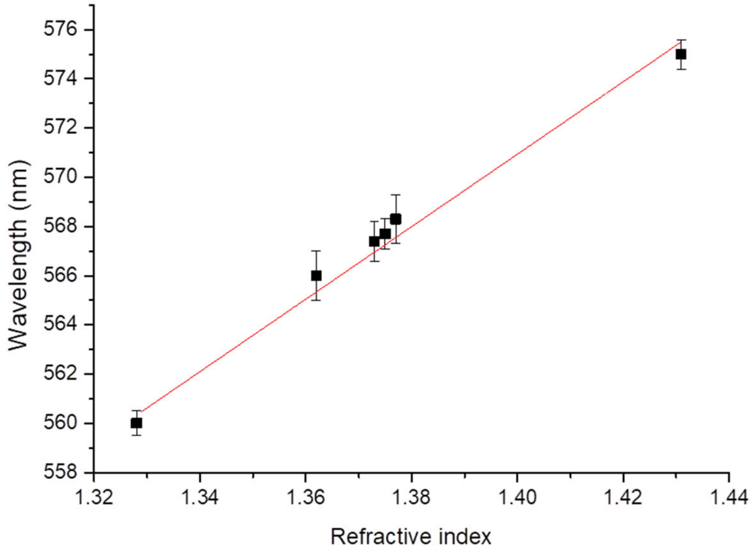

Figure S6. Plot of the wavelength shift of neat pSiRF versus refractive indices of methanol, ethanol, ethyl acetate, hexane, isopropanol and ethylene glycol infiltrated the pores, (λ(nm) = 147.38 nm × n + 364 nm, R^2=0.98). The data are presented as mean value of three measurements with the standard deviation as error. The solid red line corresponds to a linear least-squares fit of the data.

Table S1. Calculated refractive index of the void (PLA plus air) in pSiRF(dried state) from Figure S3. The refractive index shift was calculated by subtracted n values at a given time (n_t) from the n at time zero (n_{ref}).

Incubation time (min)	Refractive index of the void n_{void} (PLA+air) in pSiRF	Refractive index shift (n_t-n_{ref})
0	1.262	0.000
30	1.224	-0.038
60	1.170	-0.092
120	1.072	-0.190
240	1.011	-0.251
360	1.006	-0.256

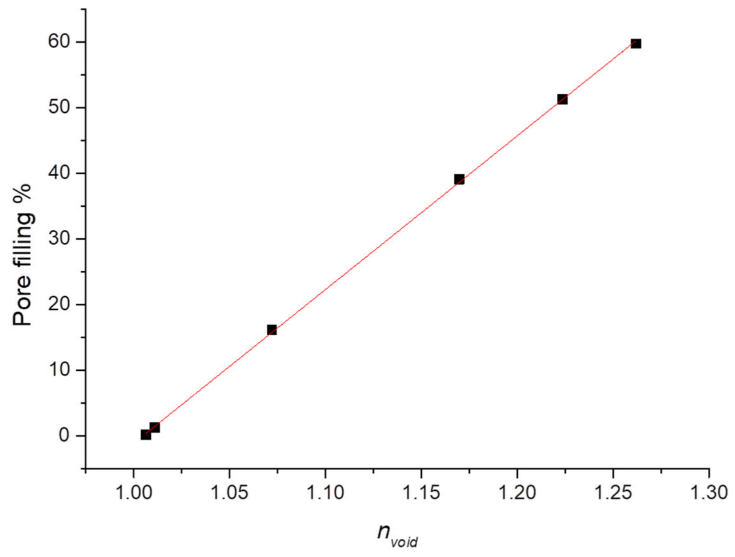

Figure S7. Plot of pore filling vs. refractive index of the voids (*filling*% = 234*n_{void}-235, R^2=0.99). The solid red line corresponds to a linear least-squares fit of the pore filling data.

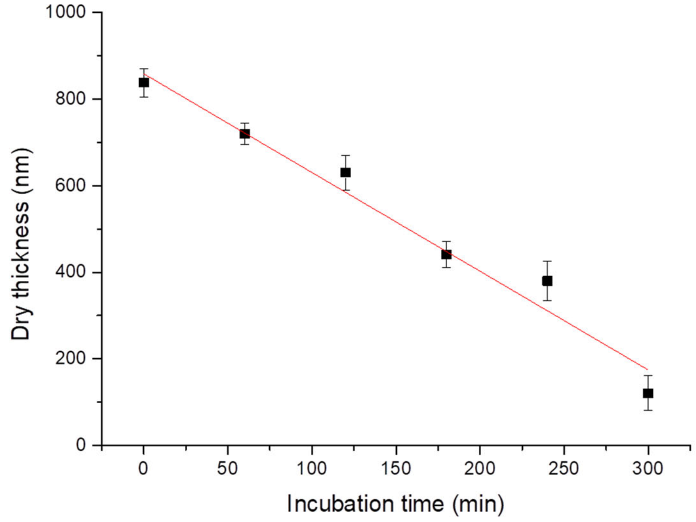

Figure S8. Dry ellipsometric thickness of PLA film on planar Si versus enzymatic degradation time in 1 mg mL^{-1} proteinase K, (D (nm)= 858 nm – 2.27 min^{-1} × t_{deg}, R^2=0.97). The data were presented as mean value of three measurements with standard deviation as error. The solid red line corresponds to a linear least-squares fit of the ellipsometric data.

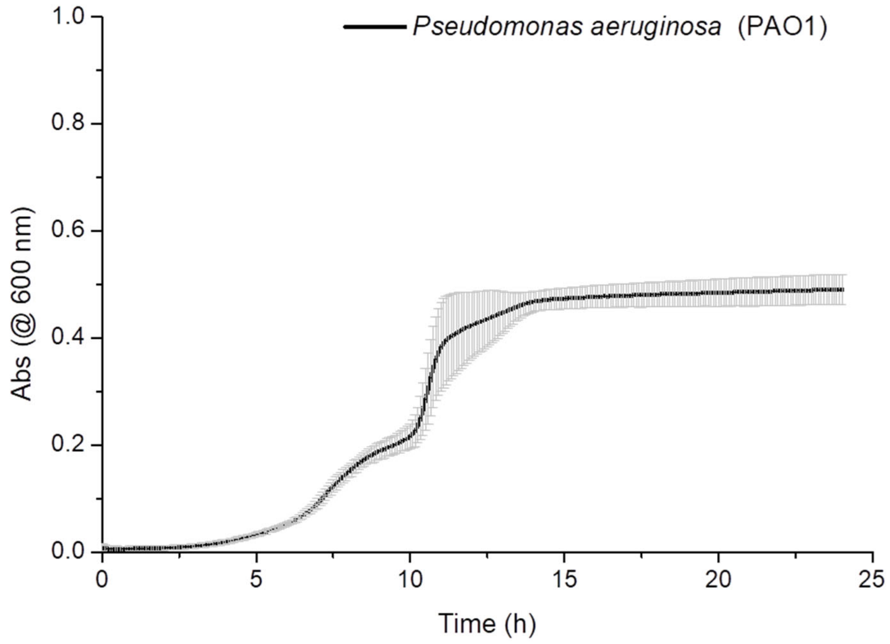

Figure S9. Bacteria growth curve of *P. aeruginosa* (PAO1) grown at 37°C, 200 rpm, for 24 h. The error bars indicate standard deviation (*n*= 3 technical replicates: filled wells with bacterial cultures).

References

1. Van Hoi Pham; Thuy Van Nguyen; The Anh Nguyen; Van Dai Pham; Huy Bui. Nano Porous Silicon Microcavity Sensor for Determination Organic Solvents and Pesticide in Water. *Advances in Natural Sciences: Nanoscience and Nanotechnology* **2014,** *5* (4), 45003. DOI: 10.1088/2043-6262/5/4/045003.

2. Salem, M. S.; Sailor, M. J.; Harraz, F. A.; Sakka, T.; Ogata, Y. H. Electrochemical Stabilization of Porous Silicon Multilayers for Sensing Various Chemical Compounds. *Journal of Applied Physics* **2006,** *100* (8), 83520. DOI: 10.1063/1.2360389.
